# Psychological risk factors of micro- and macrovascular outcomes in primary care patients with type 2 diabetes: rationale and design of the DiaDDZoB Study

**DOI:** 10.1186/1471-2458-10-388

**Published:** 2010-07-01

**Authors:** Giesje Nefs, François Pouwer, Johan Denollet, Victor JM Pop

**Affiliations:** 1 CoRPS - Center of Research on Psychology in Somatic diseases, Department of Medical Psychology and Neuropsychology, Tilburg University, Tilburg, The Netherlands

## Abstract

**Background:**

Depression is a common psychiatric complication of diabetes, but little is known about the natural course and the consequences of depressive symptoms in primary care patients with type 2 diabetes. While depression has been related to poor glycemic control and increased risk for macrovascular disease, its association with microvascular complications remains understudied. The predictive role of other psychological risk factors such as Type D (distressed) personality and the mechanisms that possibly link depression and Type D personality with poor vascular outcomes are also still unclear.

**Methods/Design:**

This prospective cohort study will examine: (1) the course of depressive symptoms in primary care patients with type 2 diabetes; (2) whether depressive symptoms and Type D personality are associated with the development of microvascular and/or macrovascular complications and with the risk of all-cause or vascular mortality; and (3) the behavioral and physiological mechanisms that may mediate these associations. The DiaDDZoB Study is embedded within the larger DIAZOB Primary Care Diabetes study, which covers a comprehensive cohort of type 2 diabetes patients treated by over 200 primary care physicians in South-East Brabant, The Netherlands. These patients will be followed during their lifetime and are assessed annually for demographic, clinical, lifestyle and psychosocial factors. Measurements include an interviewer-administered and self-report questionnaire, regular care laboratory tests and physical examinations, and pharmacy medication records. The DiaDDZoB Study uses data that have been collected during the original baseline assessment in 2005 (M_0_; N = 2,460) and the 2007 (M_1_; N = 2,225) and 2008 (M_2_; N = 2,032) follow-up assessments.

**Discussion:**

The DiaDDZoB Study is expected to contribute to the current understanding of the course of depression in primary care patients with type 2 diabetes and will also test whether depressed patients or those with Type D personality are at increased risk for (further) development of micro- and cardiovascular disease. More knowledge about the mechanisms behind this association is needed to guide new intervention studies.

## Background

The number of people with diabetes mellitus is increasing rapidly worldwide. Based on aging and other demographic changes, prevalence estimates of this chronic metabolic disease are projected to rise from 171 million in 2000 to 366 million in 2030 [1]. As the disease progresses, diabetes patients are often confronted with long-term vascular complications. While premature cardiovascular disease (including coronary artery disease, stroke and peripheral arterial disease) accounts for considerable morbidity and mortality, complications of microvascular origin also contribute significantly to adverse health outcomes [2]. To date, diabetes remains a predominant cause of vision loss, renal failure and lower extremity amputations in developed countries [3]. A large-scaled study among over 7,000 patients with type 2 diabetes in eight European countries concluded that approximately 72% of the participants had at least one complication, while 24% of the total study group had both micro- and macrovascular complications [4]. Not surprisingly, the presence of these vascular conditions has a substantial negative impact on both overall healthcare expenditures [4] and patients' quality of life [5].

### Depression is common in type 2 diabetes

Depression is another common and burdensome complication of type 2 diabetes. A recent meta-analysis of ten controlled studies showed that the prevalence of depression was significantly higher in patients with type 2 diabetes compared with non-diabetic controls (18 vs. 10%, OR = 1.6, 95% CI 1.2-2.0) [6]. Even though depression is a common co-morbidity in diabetes, little longitudinal research has been undertaken with respect to its natural course in type 2 diabetes [7]. A meta-analysis of seven prospective studies by Mezuk et al., all excluding prevalent cases of depression at baseline, concluded that the association between type 2 diabetes and the incidence of depression is only modest (RR = 1.15, 95% CI 1.02 - 1.30) [8]. However, a negative depression screening score at study entry cannot rule out a history of depression and therefore the conclusion by Mezuk et al., about the role of diabetes as a risk factor for "new" cases of depression, might be premature [9]. Two meta-analyses showed that the reversed association, with depression as a risk factor for the onset of type 2 diabetes, is stronger. Depressed adults have a 30-60% increased risk of developing type 2 diabetes [8,10].

There is abundant evidence showing that depression can be regarded as a chronic condition for many patients, with periods of (partial) remission and relapse in community [11] and primary care [12] samples. Although the existing literature suggests that depression is even more persistent in diabetes patients, these studies are hampered by relatively small numbers of type 2 diabetes patients [7,13], the inclusion of selected populations from specialised clinics [14] and the measurement of depression in a selected sample of patients who have participated in an antidepressant drug trial [13] or diabetes education programme [7,15]. A large study examining different aspects of the natural course (incidence, remission, recurrence) of depression in a representative sample of primary care patients with type 2 diabetes is currently lacking.

### Depression is associated with poor disease outcomes

Depression in diabetes was found to be associated with poor glycemic control [16], a higher number of cardiovascular risk factors [17], micro- and macrovascular complications [18], and an increased mortality risk [19-22]. Meta-analyses of prospective studies suggest that depression is associated with the onset or progression of cardiovascular disease in primary care and community samples [23] and post-myocardial infarction patients [24]. An important limitation of the current diabetes literature is that most studies on the association between depression and vascular conditions used cross-sectional data [18], hence precluding any inferences about possible causal pathways. In recent years, a limited number of prospective studies have been published. While depression predicted the incidence of vascular complications [19,25] and greater all-cause mortality [19-22], its effect on mortality due to vascular causes still is unclear [20,26]. So far, the emphasis in these studies has been on macrovascular outcomes, in particular coronary heart disease. Only two large longitudinal studies have considered the association between depression and the incidence of microvascular conditions. One of these was conducted in a sample of elderly Mexican-Americans and used self-report to ascertain the presence of complications [19], while the other only examined advanced complications, including end-stage renal disease, low vision or blindness, and amputations [27].

### Type D personality and cardiovascular disease

Most research on the psychological aspects of diabetes has focused on depression, leaving the role of other dimensions of emotional distress, such as anxiety and more stable emotional traits, as understudied areas. An emerging risk factor in the cardiovascular research domain is "Type D (distressed) personality", which is defined by the two stable personality traits "negative affectivity" and "social inhibition" [28]. Individuals with this personality type tend to experience negative emotions across time and situations, but are inclined to inhibit the expression of emotions and behaviors in order to avoid disapproval or rejection [28,29]. Type D personality is relatively common, with prevalence estimates ranging from 21% in the general population to 28% in coronary heart disease patients and 53% in hypertensives [28]. Accumulating evidence suggests that having a Type D personality is associated with a 2 to 5-fold increased risk of adverse prognosis, impaired quality of life and emotional distress across cardiovascular patient groups, independent of standard biomedical risk factors [29,30]. No studies to date have been undertaken to examine the impact of Type D personality on disease-related outcomes in patients with type 2 diabetes, although vascular disease is relatively common in this group.

### Mechanisms that could link depression and Type D with poor outcomes

Several plausible mechanisms have been hypothesized to mediate the association between emotional distress (depression, Type D) and poor vascular outcomes. Potential mediators include health behaviors, such as smoking, alcohol consumption and physical inactivity, and biomedical factors (e.g. underlying cardiac disease severity, an unfavorable cardiovascular risk profile/the "metabolic syndrome", immune processes) [31-33]. In the Heart and Soul Study, a cohort of more than 1,000 outpatients with stable coronary heart disease, the association between depressive symptoms and adverse cardiovascular events was largely explained by behavioral factors, in particular physical inactivity (32% change in effect size) [33]. The extent to which these mechanisms account for the increased risk of vascular complications in distressed diabetes patients, should this association exist, is still unclear.

### Innovative aspects of the DiaDDZoB Study

To summarize: (1) While there are numerous studies that aimed to determine the prevalence of depression in type 2 diabetes patients, little is known about the natural course of depression in diabetes (incidence, recurrence, remission). (2) The majority of studies examining the association between emotional distress and vascular disease had a cross-sectional design, focused on depression and had macrovascular disease as outcome. The role of other aspects of emotional distress and the association with common microvascular complications is therefore still unclear. (3) It is unknown which behavioral and/or biomedical mechanisms may account for the hypothesized associations between emotional distress and vascular conditions. The DiaDDZoB Study (**Dia**betes, **D**epression, Type **D **Personality **Z**uid**o**ost-**B**rabant) will examine the abovementioned issues in a cohort of primary care patients with type 2 diabetes.

## Methods/Design

### Aims and hypotheses

The DiaDDZoB Study was designed as a prospective cohort study and aims to address the following main research questions:

1. What is the natural course (prevalence, incidence, recurrence, remission) of depressive symptoms in a sample of primary care patients with type 2 diabetes?

2. Do patients with type 2 diabetes and co-morbid emotional distress (as evidenced by an increased level of depressive symptoms and/or Type D personality) have an increased risk for the onset/progression of micro- and macrovascular complications?

3. Do these types of emotional distress also increase the risk of all-cause or vascular mortality?

4. When a significant relation is found in (2) or (3): which factors mediate the association between emotional distress and diabetes outcomes?

Based on the current literature, we hypothesize the following: Approximately one fifth of our sample will have an increased level of depressive symptoms at each separate measurement occasion (prevalence). In the group of patients without a self-reported history of depression, incident depression will be low (< 5%). In the patients with a history of depression, recurrence rates will be relatively high (at least 25%). Significant risk factors for depression most likely will be: (1) psychosocial factors such as stressful life events and loneliness and (2) the presence (onset or progression) of vascular complications. We also hypothesize that patients with co-morbid distress (either depressive symptoms or Type D) will be at increased risk for the development of micro- and macrovascular conditions and both all-cause and vascular mortality; these associations are (partly) explained by behavioral (smoking behavior, alcohol consumption, physical inactivity) and biomedical (cardiovascular disease history, characteristics of the metabolic syndrome) mechanisms.

### Study design

From 2005 onwards, data for the DiaDDZoB Study have been collected within the framework of the DIAZOB (Diabetes care Zuidoost-Brabant) project, a large-scale diabetes management programme for primary care patients with type 2 diabetes. To evaluate the implementation of this standard diabetes care programme in daily practice, an observational cohort study (the DIAZOB Primary Care Diabetes study) was designed, including annual assessments of a broad range of demographic, medical, lifestyle and psychosocial factors [34,35]. Follow-up surveys of the total DIAZOB population (N ≈ 12,000) are planned for the upcoming years. The DiaDDZoB Study builds upon data from three completed measurement occasions. The original baseline measurement (M_0_) took place in the second half of 2005. Follow-up assessments were realized in 2007 (M_1_) and 2008 (M_2_).

### Subjects

The ongoing assessment of the DIAZOB-cohort is conducted in collaboration with over 200 general practitioners who are currently allied to PoZoB (Praktijkondersteuning Zuidoost Brabant), a large managed care organisation responsible for the implementation of the DIAZOB standard care programme. The practices are located in the South-Eastern area of the Netherlands, mainly in the region south of the city of Eindhoven. The patient population is residing in rural and suburban areas. To be included in the DIAZOB Primary Care Diabetes study, the patient had to be formally diagnosed with type 2 diabetes according to the guidelines of the Dutch College of General Practitioners (as evidenced by either a fasting glucose concentration of > 6.9 mmol/l in venous plasma or > 6.0 mmol/l in capillary blood on two separate days or an arbitrary glucose level > 11.0 mmol/l in the presence of the classic hyperglycemia symptoms [36]. These criteria are comparable to the recommendations of the American Diabetes Association [37]. Other inclusion criteria were: the patient was receiving treatment for diabetes in the DIAZOB diabetes care programme, had the primary care practice nurse as his/her main health care provider for diabetes issues, was at least 18 years old (with no upper age limit) and had sufficient mastery of the Dutch language. Patients were excluded if they had a treatment or condition other than type 2 diabetes as the primary cause of the hyperglycemia and/or were physically/mentally incapable of completing a questionnaire (e.g. co-morbid dementia, terminal cancer), as judged by the primary care practice nurse.

### Recruitment of patients

From 2005 onward, patients were invited by their primary care practice nurse to participate in the DIAZOB standard care project. In the period of April (pilot) and June - December 2005, the DIAZOB patients were informed of the evaluation study and received a detailed description of its practical and scientific aim. Patients who were willing to participate were asked to sign an informed consent form. Consent was sought for (a) using the anonymised data (questionnaire and medical information) for reports and scientific publications; requesting information from the patient's (b) pharmacist and (c) specialist; and (d) informing the general practitioner or primary care practice nurse of study results, if necessary.

### Participant drop-out

**Figure 1:**
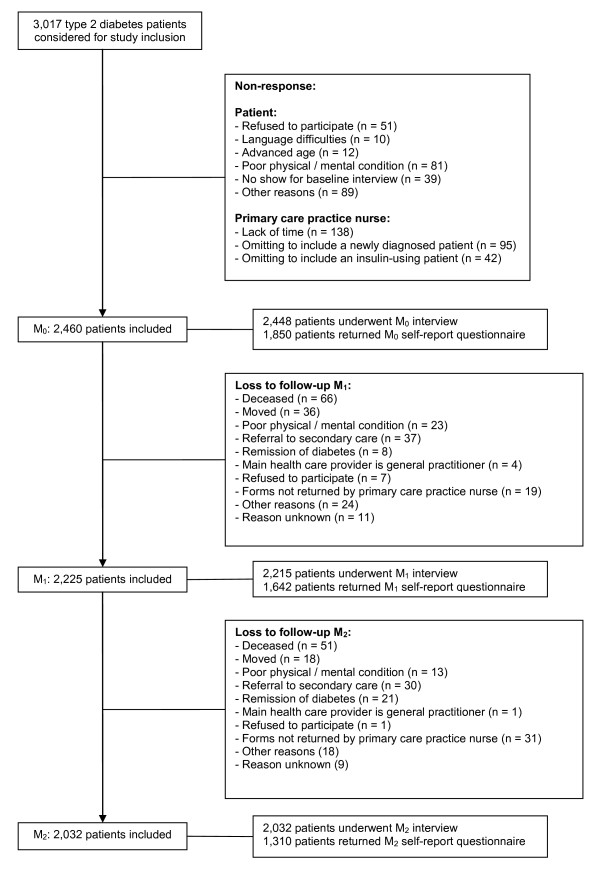
**Flow chart of the original DIAZOB cohort**.

In the beginning of 2005, the total number of type 2 diabetes patients in the area covered by the participating general practitioners at that time was estimated at 3,000 to 3,500. During the baseline inclusion period, 3,017 patients were considered for participation in the study. A detailed overview of the study's participation and drop-out rate can be found in Figure 1. Reasons for baseline non-response could be grouped into "patient characteristics" (e.g. not meeting inclusion criteria/screening positive on exclusion criteria, refusing to participate, not showing up at the baseline interview) and "practice nurse characteristics" (lack of time, omitting to invite newly diagnosed or insulin-using patients). Of the resulting 2,460 patients, 2,448 (99.5%) attended the interview and 1,850 (75.2%) returned the self-report questionnaire that had to be filled in at home. For the M_1 _and M_2 _assessments, 2,225 and 2,032 patients were available, respectively.

### Measures used in the DiaDDZoB Study

The DIAZOB Primary Care Diabetes study measurements include an interviewer-administered and self-report questionnaire, results from regular care laboratory tests and physical examinations, and pharmacy medication records. An overview of the variables that were used for the DiaDDZoB Study can be found in Table 1.

**Table 1:** Measurements included in the DiaDDZoB Study

Variable	Categories	Measurement occasion
Demographic factors		
Age		M_0_
Gender	Male, female	M_0_
Ethnicity	Dutch, other Caucasian (white Western) groups, other (Asian, black, Turkish/Moroccan)	M_0_
Marital status	Married/living together, single, LAT relationship, divorced/ separated, widowed	M_0_, M_1_, M_2_
Living situation	Independent, residing with family/friends, residing in a nursing home	M_0_, M_1_, M_2_
Employment status	Paid employment, umemployed, disabled, no paid employment, retired	M_0_, M_1_, M_2_
Education level	Primary school, primary vocational education, secondary school, secondary vocational education, higher vocational education, university	M_0_
Medical history		
Disease duration	Months/years since diabetes diagnosis	M_0_, M_1_
Medical history	M_0_: lifetime history: arterial disease, bypass/angioplasty, myocardial infarction, stroke, angina pectoris, high cholesterol, kidney disease, asthma/COPD, cancer, rheumatic disorder, depression, burn-out M_1 _and M_2_: during last 12 months: arterial disease, bypass/angioplasty, myocardial infarction, stroke, angina pectoris, high cholesterol, asthma/COPD, osteoporosis, depression, kidney disease	M_0_, M_1_, M_2_
Treatment		
Current hyperglycemia treatment modality	None, diet, diet/oral agents, diet/ insulin, diet/oral agents/insulin, other	M_0_, M_1_, M_2_
Medication use	ACE inhibitors, β-blockers, calcium antagonists, diuretics, other antihypertensive agents, statins	M_0_, M_1_, M_2_
Laboratory tests		
HbA_1c_		M_0_, M_1_, M_2_
Fasting glucose		M_0_, M_1_, M_2_
Cholesterol	Total cholesterol	M_0_, M_1_, M_2_
	LDL-cholesterol	M_0_, M_1_, M_2_
	HDL-cholesterol	M_0_, M_1_, M_2_
	Triglycerides	M_0_, M_1_, M_2_
Protein levels	Albumin	M_0_, M_1_, M_2_
	Creatinin	M_0_, M_1_, M_2_
	Albumin-to-creatinin ratio	M_0_, M_1_, M_2_
	MDRD clearance	M_1_, M_2_
Physical examination		
Length	Length in metres	M_0_, M_1_, M_2_
Weight	Weight in kilograms	M_0_, M_1_, M_2_
Body Mass Index (BMI)	Weight in kilograms/(length in metres)^2^	
Blood pressure	Systolic	M_0_, M_1_, M_2_
	Diastolic	M_0_, M_1_, M_2_
Fundus photography	Unassessable, normal, retinopathy	M_0_, M_1_, M_2_
Foot examination	M_0_: Normal, abnormal M_1 _and M_2_: Normal, neuropathy, ischemia, wound/ulcer, excessive coldness	M_0_, M_1_, M_2_
Lifestyle indicators		
Smoking behavior	Current smoking: yes/no, number of cigarettes per day	M_0_, M_1_, M_2_
	Additional for M_0_: smoking history	
Alcohol consumption	Current alcohol consumption: yes/no, number of consumptions per week	M_0_, M_1_, M_2_
	Additional for M_0_: history of alcohol consumption	
Physical activity	Hours per week of active physical activity	M_0_, M_1_, M_2_
	Hours per week of sportive physical activity	M_0_, M_1_, M_2_
Psychological factors		
Depressive symptoms	Edinburgh Depression Scale (EDS)	M_0_, M_1_, M_2_
Type D personality	Type D Scale-14 (DS14)	M_1_, M_2_
Social support	O'Hara's modified Social Support Scale	M_0_, M_1_, M_2_
Loneliness	Single item concerning feelings of loneliness in the past 12 months	M_0_, M_1_, M_2_
Stressful life events	Single item concerning stressful life event(s) in the past 12 months	M_0_, M_1_, M_2_

#### Data acquired during an interview with the primary care practice nurse

The interview-administered questionnaire was filled out by the practice nurse along with the patient during regular diabetes check-up and included questions about demographic factors, clinical parameters, hyperglycemia treatment and health behaviors.

##### Demographics

Information was gathered about age, gender and ethnicity.

##### Clinical parameters

At baseline, the primary care practice nurse recorded the number of months/years since diabetes diagnosis and took a basic medical history, including self-reported history of depression. Self-reported medical diagnoses were verified through inspection of the medical record. At follow-up, onset and/or progression of vascular complications and other conditions was recorded, as was mortality date and cause of death for those patients who deceased during the study.

##### Hyperglycemia treatment

At each measurement occasion, the practice nurse documented whether patients were currently treated for their hyperglycemia by diet, oral agents, insulin or a combination of these treatment modalities.

##### Health behaviors/Lifestyle

A basic overview of current and former smoking behavior and alcohol consumption was collected at the baseline assessment. Changes to this baseline pattern were recorded during follow-up. Physical activity was assessed by means of two items. Patients had to indicate how many hours per week they spent on (a) "active" (all physical activities other than practicing sports, e.g. gardening, walking, cycling, climbing stairs) and (b) "sportive" physical activities (e.g. sports, fitness).

#### Data acquired using self-report questionnaires

A second questionnaire was completed by the patient at home and addressed several additional demographic variables and psychosocial factors. For practical purposes, the questions about health behaviors were transferred from the interview-administered to the self-report questionnaire for the follow-up measurements.

##### Demographics

The demographic measures included marital status (dichotomized as being single versus having a partner), living situation (independent versus dependent of others), employment status (paid employment versus no paid employment/unemployed/disabled/retired) and educational level (low education versus middle/high education).

##### Depressive symptoms

Depressive symptoms during the last seven days were assessed using a validated Dutch version of the Edinburgh Depression Scale (EDS) [38]. This is a 10-item self-rating scale in which each item is scored on a four-point scale. Total scores range from 0 to 30 points. The EDS was originally developed to measure post partum depression [39], but has later been validated in non-postnatal women [40], women around menopausal age [41], men [42] and community samples [43]. Although cut-off points for predicting a diagnosis of clinical depression vary [44], a cut-off score of 12 or more seems to have satisfactory sensitivity and specificity [41,43].

##### Type D personality

Type D personality was assessed using the Type D Scale-14 [28]. This questionnaire consists of 14 items, which are scored on a five-point rating scale ranging from 0 = "false" to 4 = "true". The DS14 comprises two scales, one measuring level of negative affectivity (NA) and the other social inhibition (SI). Subjects who obtain a score of ten or more on both scales are considered to have a Type D personality [28]. Both scales have been shown to be internally consistent (Cronbach's α = 0.88 for the NA scale and 0.86 for the SI scale), stable over an 18-month period [45] and are independent of mood and health status [28,45].

##### Other psychosocial factors

Social support was measured using O'Hara's modified Social Support Scale [46], comprising three items. Answer categories range from 0 to 4 points, with 0 indicating "no social support at all" and 4 indicating "extensive social support". The total social support score is obtained by adding scores on all three items. A single item was used to measure feelings of loneliness in the past 12 months, which were scored on a scale from 1 to 10 points, with a score of 1 meaning "I never felt lonely" and a score of 10, "I always felt lonely". To account for non-diabetes related stressors, respondents were asked if they had experienced a stressful life event in the previous 12 months (e.g. loss of a loved one, a break-in, relationship problems, loss of work, serious financial problems, physical/mental abuse).

#### Laboratory tests and physical examinations

Biomedical parameters were derived from standard care laboratory tests and physical examinations carried out by the Diagnostic Centre Eindhoven, a primary care institution where biological records of the regional diabetes population are filed after each regular care check-up appointment. For the DIAZOB project, blood was drawn annually to determine glycemic control (glycosylated hemoglobin or HbA_1C _levels, fasting glucose), creatinin, the MDRD clearance (only at follow-up) and cholesterol values (total, LDL, HDL, triglycerides); urine samples were taken to assess albumin and the albumin-to-creatinin ratio. As for the physical examination, blood pressure measurements (systolic and diastolic), body mass index (BMI; weight in kilograms/length in metres^2^), and fundus photography and foot screening results were provided. To diagnose retinopathy, digital fundus photography was carried out by a biometrist and interpreted by an ophthalmologist. Foot screening included a neurological and vascular examination (Doppler test), and inspection of feet and shoes by a podotherapist or a biometrist under supervision. For the baseline measurement, only the presence of abnormalities to the feet was recorded. During follow-up testing, the lower extremities were assessed for neuropathy, ischemia, wounds/ulcers and excessive coldness.

#### Pharmacy medication records

With the patient's consent, information regarding prescribed medication was obtained from local pharmacists. In addition to the medication applied for the management of hyperglycemia, the use of cardiovascular agents (including several antihypertensives and a class of cholesterol-lowering drugs) was registered.

### Ethical principles

This study was planned and conducted in accordance with the medical professional codex and the Helsinki Declaration of 1996 [47]. Written informed consent was obtained from all participants. The study protocol of the DiaDDZoB Study was approved by the medical research ethics committee of a local hospital, the Máxima Medical Centre in Veldhoven (NL27239.015.09).

### Planned statistic analyses

Statistical analyses will be performed using the latest version of the Statistical Package for Social Sciences (SPSS). A p < 0.05 significance level will be adopted in all statistical tests. As the number of previous studies on these research topics is limited, we choose to use two-sided tests in all analyses.

Frequencies will be provided for (1) the prevalence, (2) incidence (with/without self-reported history of depression), (3) recurrence (high score across two or three assessments) and (4) other patterns of relapse and remission of high depressive symptoms (EDS-score of 12 or more). In addition, logistic regression analyses will be used to determine significant predictors of these different course patterns. Baseline characteristics of patients with/without high depressive symptoms (EDS-score of 12 or more) and with/without a Type D personality will be compared using independent-samples t-tests and X^2 ^tests. To evaluate the vascular risk associated with increased levels of emotional distress, we will perform logistic regression analyses for (1) the development of each separate micro- and macrovascular complication and (2) a composite measure of vascular disease (the development of any vascular condition) during the two year follow-up period, with either depression or Type D personality as the independent variable. The group of participants with low depressive symptoms or no Type D personality, respectively, will be used as the reference category. Analogous analyses will be used for mortality, with the dependent variable defined as (1) all-cause mortality or (2) (cardio)vascular mortality, as registered in primary care medical records up until December 2008. Before proceeding to the multivariate statistics, several study variables will be evaluated for their potential as confounders or mediators in the association between emotional distress and disease outcomes (the onset/progression of micro- and macrovascular complications, all-cause and vascular mortality). In line with the methods used in a study by Whooley et al. [33], we will adopt a > 5% change in the effect size (odds ratio) for emotional distress before and after adjustment for the variable in question as the criterion to identify suitable mediating or confounding factors. All variables satisfying these conditions will be included in the final logistic regression models. In addition, we will look at mediating variables more closely using one of the statistical methods described in the recent article by MacKinnon, Fairchild and Fritz [48].

### Power calculation

The sample size was determined using PASS 2008 [49] and was based on the logistic regression analyses for the main research question ("Do patients with type 2 diabetes and co-morbid emotional distress have an increased risk for the onset/progression of micro- and macrovascular complications?"). Assuming a power of 0.80, an alpha level of 0.05, two-sided testing, and a baseline prevalence rate of 20% for the binary independent variable (either high levels of depressive symptoms or Type D personality), we calculated the sample size for a range of scenarios. Based on earlier primary care and community studies [19,27] and on known characteristics of the DIAZOB population, we expect a two-year cumulative event rate (the development of any vascular complication) of 10 - 15%. In psychological research, R^2 ^(achieved when emotional distress is regressed on the other independent variables) usually ranges from 0.20 - 0.30. Assuming equivalence between OR/RR/HR due to the relatively low event rate of vascular outcomes, the majority of earlier studies on the risk of vascular disease in diabetes patients, primary care/community samples and post-myocardial infarction patients has found an effect size for depression of approximately 1.5 - 2.0 [23,24,27]. Therefore, the entered values were either 0.10, 0.125 or 0.15 for P_0 _(the probability that a participant develops any vascular complication during the two year follow-up period, given that he/she has a low level of depressive symptoms or no Type D personality at baseline), 0.20, 0.25 or 0.30 for R^2^, and 1.5, 1.75 or 2.0 for the OR. Sample sizes ranged from 3905 in the most conservative scenario (P_0 _= 10, R^2 ^= 0.30 and OR = 1.5) to 764 in the least restricted scenario (P_0 _= 15, R^2 ^= 0.20 and OR = 2.0). When positing a middle-ground scenario (P_0 _= 0.125; R^2 ^= 0.25 and OR = 1.75), the study needs a total of 1499 participants to detect an OR of 1.75. Anticipating an annual 10% loss to follow-up (death, serious illness, moving), we need to include approximately 1850 patients at baseline. As we expect a 40% non-response/exclusion rate, we will consider for eligibility the total patient group (n ≈ 3000).

## Discussion

As the prevalence of type 2 diabetes is high and the absolute numbers of patients with both diabetes and depression will continue to rise considerably in the next decades, it has become even more essential to further increase our understanding of the associations between diabetes and depression. Currently, our knowledge about this area is still limited. The present paper gives an outline of the theoretical background and methodology of the DiaDDZoB Study, a Dutch prospective cohort study in primary diabetes care, which aims to answer several key research questions regarding the course and vascular impact of depression and Type D personality in patients with type 2 diabetes.

The major strengths of the DiaDDZoB Study include its longitudinal design and relatively large sample size, its focus on type 2 diabetes patients who are being treated in a primary care setting, the wealth of detailed patient information that is available, and the policy to verify self-reported disease by inspection of medical records. The results of this study may lead to the identification of high risk patients and could guide the development of future intervention studies.

While the DIAZOB Primary Care Diabetes study aims to include a relatively unselected patient population, depressive symptoms (including a depressed mood or markedly diminished interest, loss of energy and a diminished ability to think or concentrate) could have negatively affected the initial decision to participate in those patients who would otherwise have screened positive for depression on the EDS. In a similar vein, as the nurse-led interview required a certain amount of openness and direct communication with a health provider about disease and concurrent complaints, social inhibition might have deterred patients with a Type D personality. In a study of 178 patients with chronic heart failure, patients with a Type D personality not only experienced and worried more about cardiac symptoms, they also were less likely to report these symptoms to their cardiologist or nurse [50].

The sharp rise in the number of patients with type 2 diabetes has resulted in a gradual shift from secondary to primary care, thereby placing considerable demands on primary health care teams [51]. Secondary care consultations or referrals are indicated in case of more complex disease management, e.g. in the presence of complications or poorly regulated blood glucose levels [52,53]. Seeing that approximately 3% (67/2,460) of all patients participating in the baseline assessment dropped out of the study after a secondary care referral, future assessments should be planned and carried out in close cooperation with hospital practitioners to keep the cohort intact. To alleviate some of the workload for general practitioners, the primary care nurse specialist has been introduced as the main care-provider for patients with type 2 diabetes in family practice [54]. Since nurse practitioners generally provide longer consultations and are trained to focus on the medical, practical as well as the emotional aspects of diabetes [55], depression prevalence estimates could have been lowered with more adequate detection and subsequent treatment of depression. Unfortunately, earlier work has shown that the reverse is probably true, i.e. the presence of emotional problems was recorded in the medical chart in only 20 - 30% of diabetes patients with high scores on questionnaires measuring emotional distress [55].

Although a recent cohort study has investigated whether depression runs a chronic course with high rates of recurrence in primary care [12], there is a paucity of research on the trajectory of emotional distress in specific chronic diseases. While our annual screening method most likely will identify a subsample with recurrent, high levels of depressive symptoms, a prospective design with yearly follow-up assessments will by definition miss some patients with a relapsing-remitting symptom profile who happen to be in complete or partial remission at the assessment occasion. While an in-depth characterization of short-term fluctuations in emotional distress goes beyond the initial goals of the DIAZOB project, a similar model offers interesting research perspectives for future studies.

A structured psychiatric interview using DSM-IV criteria is considered the gold standard for diagnosing clinical depression [56]. While self-report questionnaires were originally developed to quantify the severity of depression, they are often adopted as time-efficient case-finding instruments in large samples [57]. However, depression is not the only common emotional problem in diabetes patients [58,59]. Depression questionnaires may detect some [56], but certainly not all [58] components of distress. Given the tendency of psychosocial factors to cluster together within individuals [60,61], the simultaneous assessment of multiple distress types seems justified to obtain a more precise risk stratification [60]. Other studies have emphasized the importance of examining both episodic emotional states and more chronic psychosocial factors [60,62]. By extending the focus of our research from a cross-sectional assessment of depressive symptoms to repeated measurements of not only depression, but also Type D personality, we intend to take a step in this direction.

Several other study limitations need to be mentioned. First, while laboratory determinations and physical examinations are part of regular care protocols and therefore did not impose an extra burden on the participating patients, we cannot avoid that for some patients these tests were scheduled several months before or after the official measurement occasion in question. In these cases, we used the test results that were the closest in time to the rest of the data. Secondly, although the prescription of antihyperglycemic and cardiovascular agents was documented relatively well, information on the concurrent use of psychotropic medication is lacking. Earlier studies have suggested that different classes of antidepressant drugs may exert a clinically relevant positive or negative effect on glucose-insulin homeostasis [63]. Requesting pharmacy information from large databases of pharmacy dispensing records [64] might improve the accuracy of medication registration. Finally, although we aim to elucidate the mechanisms responsible for the adverse effect of emotional distress on vascular outcomes, no information was available on several interesting candidate mechanisms, including dysfunctional activity of the hypothalamic-pituitary-adrenal axis, neurotransmitter function or inflammatory processes [31-33].

General practice settings offer relatively favorable conditions for conducting longitudinal research, as the Dutch health care system is characterized by a high level of care continuity between patients and family physicians [65]. In enhancing the quality of longitudinal data collection, it is essential to ensure the provision of solid research facilities, standardization of data between practices and over time, and integration of scientific and patient care data collection in a clinician-friendly manner [65]. Planning and conducting longer term epidemiologic studies considerably challenges the motivation and benevolence of participating health care providers. Apart from central coordination of the DIAZOB research infrastructure, PoZoB also contributes to a clinical translation of scientific findings by organising feedback meetings and training programmes, thus enabling an ongoing research commitment of general practitioners and their staff [65]. Keeping an eye on the needs and developments in primary care daily practice, the collaboration between PoZoB and C*o*RPS, Tilburg University, will provide an excellent framework to explore the wealth of information already available and at the same time ensure a continuing qualitative and innovative development of primary care diabetes research.

## Competing interests

The authors declare that they have no competing interests.

## Authors' contributions

All authors have contributed to the design and content of this study; all authors have read and approved the final manuscript.

## Pre-publication history

The pre-publication history for this paper can be accessed here:

http://www.biomedcentral.com/1471-2458/10/388/prepub
